# 1374. Clinical Outcomes of Sepsis According to Race at University of Minnesota Medical Center

**DOI:** 10.1093/ofid/ofab466.1566

**Published:** 2021-12-04

**Authors:** Cameron Meyer-Mueller, Darlisha A Williams, Michael Westerhaus, Radha Rajasingham

**Affiliations:** 1 University of Minnesota, Minneapolis, Minnesota; 2 University of Minnesota; Center for International Health; EqualHealth, Minneapolis, Minnesota

## Abstract

**Background:**

Sepsis is a life-threatening condition associated with significant in-hospital mortality. Sepsis disproportionately affects Black Americans and is a top-10 leading cause of death for Black people. Previous studies examining sepsis mortality rates by race have yielded inconsistent findings. This retrospective study evaluates the relationship between race and in-hospital sepsis-related mortality in adults at University of Minnesota Medical Center.

**Methods:**

We reviewed all sepsis diagnoses in adults between January 1, 2020 and June 30, 2020 at the University of Minnesota Medical Center. Demographic information including age, sex, race, insurance status, primary language, expected and observed mortality score, discharge status, treatment information, and in-hospital mortality were also recorded. Self-reported race was categorized as African American, White, American Indian or Alaska Native, Asian, African, Hispanic or Latino, Hawaiian or other Pacific Islander, “some other race,” and “two or more races.” Statistical tests including χ  2 test, Student t test, Kaplan-Meier estimator, and binary logistic regression were performed.

**Results:**

We identified 780 cases of sepsis. Black patients were consistently younger than White patients (median age of 50 years, compared to 61 years, p< 0.001). Black patients were more likely to have comorbidities at baseline. However, logistic regression analyses, after controlling for language, race, primary payer, and expected mortality, showed no association between sepsis outcome and race.

Sepsis Cases at UMMC between January and June 2020 by Self-Reported Race

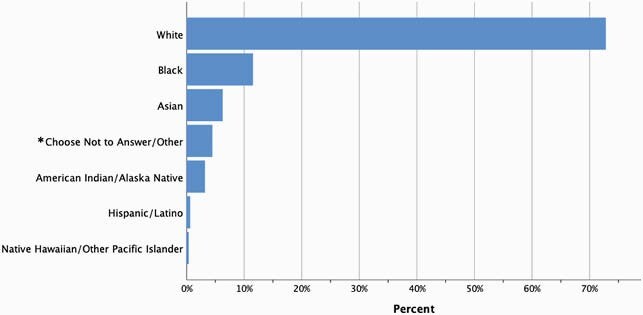

*Other includes the categories “Some other race” and “Two or more races.”

Hospital Outcomes by Race

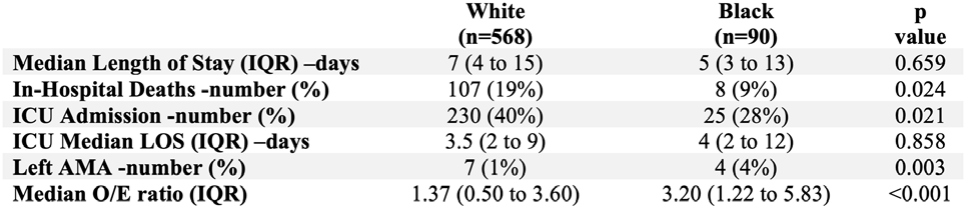

Patient Demographics by Race

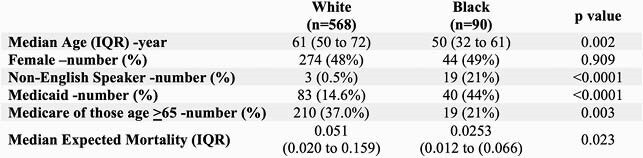

**Conclusion:**

While there was no significant difference between in-hospital mortality and race, Black patients were more likely to present at a younger age with more medical comorbidities than White patients.

**Disclosures:**

**All Authors**: No reported disclosures

